# The Relationship between Substance Use and HIV Transmission in Peru

**DOI:** 10.4172/2155-6105.1000129

**Published:** 2012-07-22

**Authors:** Alfredo A. Massa, Marc I. Rosen

**Affiliations:** 1 Department of Psychiatry, Yale University School of Medicine, Chacarilla-Surco, Lima-Peru, USA; 2 Department of Psychiatry, Yale University School of Medicine, VA Connecticut Healthcare System, West Haven, USA

**Keywords:** HIV transmission, Substance-related-disorders, Peruvian research, Peru

## Abstract

**Objectives:**

The primary aim of this article is to review literature regarding the relationship between substance use and HIV transmission in Peru.

**Methods:**

Detailed search of published literature completed in PubMed and Google-Scholar and other local Peruvian publications. Mesh words: “Peru”; “substance-related-disorders”; “HIV”; “sexual-behavior” and their combinations. From 3921 articles, 150 were chosen for more careful review and only 26 were used for the review. No date limit was used in this review.

**Results:**

Peruvian HIV epidemic is limited to MSM and its prevalence goes up to 33% in certain MSM-subpopulations. Transmission is mainly through sexual contact. Drug use doubled the risk for casual sex, decreased by half the chances of using condoms, increased the number of partners per year and the risk for STD’s. Peruvian HIV-positive populations have higher rates drug use and using drugs have been associated with a higher prevalence of being HIV-positive. This may be also true for other populations such as pregnant women in which there is an association between drug use and HIV.

**Conclusions:**

Although the amount of Peruvian research in this area limits the review, there seems to be a relationship between using drugs, having risky-sexual-behaviors and being HIV positive in Peru. HIV-prevention strategies for Peruvians must address the link between sex and substance use.

## Brief Overview of the Peruvian HIV Epidemiology and Population at Risk of HIV in Peru

The first case of Acquired Immune Deficiency Syndrome (AIDS) in Peru was reported in 1983 [1] and as April 2010 the Ministry of Health (MoH) had identified almost 38867 cases of human immunodeficiency virus (HIV) and 25666 of AIDS [2]. However, the number of estimated cases may be higher [1]. The World Health Organization (WHO) defines a concentrated HIV epidemic, as a situation in which high risk groups have an HIV prevalence of 5% or more [3]. This is the case in Peru where the prevalence of HIV in Men who have sex with Men (MSM) is greater than 5% [4]. HIV prevalence in female sex workers is between 1% and 2% in Peru, while less than 1% in the general population [5–7]. Almost 70% of HIV infected people reside in Lima [7].

Overall, 97% of cases were apparently acquired via sexual transmission (the other 3% correspond to vertical transmission and parenteral transmission) [1]. More than half of all cases are due to sex between men [7]. The low prevalence of parenteral drug use is primarily due to the almost negligible rates of injection drug use in Peru [8]. The prevalence of HIV is higher among men between 25 and 34 years of age than among older men [2].

The prevalence of HIV among MSM has been found to be above 5% [2]. But MSM are not a homogenous group and certain characteristics can greatly heighten or attenuate their risk for HIV. For example, a study reported that the prevalence of HIV among a subgroup of MSM that identified themselves as transvestites was as high as 30%. This was followed by men who identified themselves as homosexual or gay with a prevalence of 18% and men who identified themselves as bisexuals with a prevalence of 15% [9]. An important study found that 13% of heterosexually identified males who attended a sexually transmitted disease (STD) clinic with their stable female partner reported having had sexual contact with other men. This study also reported that heterosexually identified MSM were at higher risk of acquiring an STD and engaging in risky behaviors (higher number of sexual partners, anal sex and having sex for money) [10]. Sexual roles are also important in Peruvian MSM. Versatile MSM (insertive and receptive anal sex) were at the highest risk of acquiring HIV through unprotected anal sex and of transmitting the virus through insertive anal sex, even after correcting for the different frequencies of sexual encounters in the other groups (passive or receptive anal sex and active or insertive anal sex)[11].

## Overview of Alcohol-Illicit Drug use and Associated Risky Behaviors in Peru

The 2006 Peruvian Drug Use Survey shed light on the epidemiology of drug use in Peru and some of its associated factors. The survey was conducted as a face-to-face interview by trained personnel. People interviewed included a range of individuals from 12 to 64 years of age from 43 Peruvian cities.

The Survey found that alcohol is widely used with a lifetime prevalence of 83%. Of those who used alcohol in the last year, approximately 8% were dependent on alcohol and 16% abused alcohol. These numbers show the wide acceptability and accessibility of alcohol in the general population [12].

A metropolitan epidemiological study in 2002 [13] showed that alcohol was the most commonly abused drug in Peru. Marijuana was the second-most commonly abused drug with a lifetime prevalence of 3.6%, and cocaine was third with a lifetime prevalence of 2.8%. Cocaine is mainly used intranasally (cocaine hydrochloride-powder form) or smoked (coca-paste) and both cocaine and marijuana are used mainly by men [13].

Among Peruvian, heavy episodic drinking and sex-related expectations about alcohol use were associated with risky sexual behaviors. For example, young males believe that alcohol has a positive effect on their sexual performance in that it helps them and their partners to enjoy sex more. In this survey, heavy episodic drinking was associated with having had multiple sexual partners or a casual partner in the last year and not using a condom during the sexual encounter [14]. There is also evidence showing that young adults in Lima perceive the risk of acquiring HIV as low [15].

Other Peruvian studies have shown that people who use substances before sex are at particularly high risk of either having sex with another man, having unprotected sex with a casual partner, or having had an STD [16–18]. Similarly, heavy drinking significantly increases the risk of having multiple sexual partners, having had sex with a casual partner in the last year, and acquiring an STD [14,19,20]. Also, and of great importance, illicit drug use doubled the probability of intercourse with a casual partner in the last year and reduced the probability of condom use at last intercourse in half [21].

Therefore, in Peru people who use substances are at increased risk in two ways. Substance users are more likely to have multiple and unknown sexual partners who may have HIV and other STD’s, and they are less likely to use condom protection during their encounters.

## Evidence of the Association between Substance Use and HIV Transmission

Previously, we discussed the relationship between risky behaviors and drug use. In this section we will discuss the relationship between having a diagnosis of HIV, risky behaviors and drug use in Peru.

Several studies have added to the literature regarding the relationship between using drugs and being HIV-positive. For example, subjects who reported using cocaine and having had sex with men in the past year had twice the risk of being HIV-positive as the control group, even after adjusting for age, educational level, condom use and number of male sex partners [22]. The association also goes in the other direction. Peruvian studies on the HIV-positive population have reported much higher rates of substance use than those in the general population [23].

A more recent case-control study evaluated the association between alcohol addiction and HIV diagnosis. After excluding patients with a history of illegal drug use, the authors found an association between having a diagnosis of HIV and alcohol dependence. They also found that people who were HIV-positive were more likely to be homosexual, have a low income, have been born in Lima, and to have had sex with violence or in groups [24].

Studies in other populations have noted an association between drug and/or alcohol use and HIV infection. For example, a case control study designed to determine the role of sexual networks and other risk factors among HIV-positive pregnant women recruited at a perinatal treatment clinic found that a history of illegal drug use was associated to being HIV positive. Also, the male partners of these HIV positive pregnant women, who were also HIV positive, were more likely to have used illegal drugs [25]. Alcohol intoxication at least once per month and illegal drug use were associated with increased risk of acquiring HIV in another group of pregnant women in Lima [26]. The importance of women partner’s sexual network was highlighted by the finding that the likelihood of being HIV positive increased when a woman’s sexual partner either had AIDS, used illegal drugs or alcohol, used intravenous drugs, or had sex with men [26].

At this point in Peru there is some evidence of a relationship between the use of alcohol, illegal drugs and HIV. Even though more research is needed in this area, the weight of the evidence so far is that substance use contributes to the spread of HIV infection. Therefore, HIV risk reduction should be an important component of substance abuse treatment.

## Abbreviations

AIDS: Acquired Immune Deficiency Syndrome
ARV: Anti Retro Virals
HIV: Human Immunodeficiency Virus
MSM: Men Who Have Sex With Men
STD: Sexually Transmitted Disease
UNAIDS: The Joint United Nations Programme on HIV/AIDS
